# Interspecies Variation in the Functional Consequences of Mutation of Cytochrome *c*

**DOI:** 10.1371/journal.pone.0130292

**Published:** 2015-06-18

**Authors:** Tracy M. Josephs, Moira E. Hibbs, Lily Ong, Ian M. Morison, Elizabeth C. Ledgerwood

**Affiliations:** 1 Department of Biochemistry, Otago School of Medical Sciences, University of Otago, Dunedin, New Zealand; 2 Department of Pathology, Dunedin School of Medicine, University of Otago, Dunedin, New Zealand; The University of Texas MD Anderson Cancer Center, UNITED STATES

## Abstract

The naturally occurring human cytochrome *c* variant (G41S) is associated with a mild autosomal dominant thrombocytopenia (Thrombocytopenia Cargeeg) caused by dysregulation of platelet production. The molecular basis of the platelet production defect is unknown. Despite high conservation of cytochrome *c* between human and mouse (91.4% identity), introducing the G41S mutation into mouse cytochrome *c* in a knockin mouse (*Cycs* ^G41S/G41S^) did not recapitulate the low platelet phenotype of Thrombocytopenia Cargeeg. While investigating the cause of this disparity we found a lack of conservation of the functional impact of cytochrome *c* mutations on caspase activation across species. Mutation of cytochrome *c* at residue 41 has distinct effects on the ability of cytochrome *c* to activate caspases depending on the species of both the cytochrome *c* and its binding partner Apaf-1. In contrast to our previous results showing the G41S mutation increases the ability of human cytochrome *c* to activate caspases, here we find this activity is decreased in mouse G41S cytochrome *c*. Additionally unlike wildtype human cytochrome *c*, G41S cytochrome *c* is unable to activate caspases in Xenopus embryo extracts. Taken together these results demonstrate a previously unreported species-specific component to the interaction of cytochrome *c* with Apaf-1. This suggests that the electrostatic interaction between cytochrome *c* and Apaf-1 is not the sole determinant of binding, with additional factors controlling binding specificity and affinity. These results have important implications for studies of the effects of cytochrome *c* mutations on the intrinsic apoptosis pathway.

## Introduction

Thrombocytopenia Cargeeg (THC4; OMIM 612004) is one of two autosomal dominant thrombocytopenias associated with the only known mutations of the human cytochrome *c* gene (*CYCS*) [1,2]. Cytochrome *c* is an essential electron carrier in the mitochondrial respiratory chain and is also a critical mediator of the intrinsic apoptosis pathway. In response to numerous apoptotic stimuli cytochrome *c* is released from mitochondria and binds to the apoptotic protease activating factor-1 (Apaf-1) in the cytosol. Seven cytochrome *c*-Apaf-1 complexes form the apoptosome, a caspase-activating platform [3,4]. The Thrombocytopenia Cargeeg mutation results in substitution of glycine 41 by serine in cytochrome *c* (G41S cytochrome *c*). The G41S mutation has no impact on the activity of cytochrome *c* as a mitochondrial electron carrier but enhances both caspase activation and peroxidase activity [1,5,6].

The molecular basis of the abnormality in platelet production in Thrombocytopenia Cargeeg is unclear. Analyses of bone marrow electron micrographs and *in vitro* platelet production suggest accelerated platelet production resulting in platelet release into the bone marrow space instead of the circulation [1,7]. Some evidence supports the role of the intrinsic apoptosis pathway in platelet formation [8,9], while others demonstrate that, at least in mice, the apoptotic pathway needs to be restrained for effective platelet formation [10–12]. Thrombocytopenia Cargeeg subjects are heterozygous for the G41S mutation. We therefore hypothesized that a more pronounced phenotype would be observed when two copies of the allele are present. To test this hypothesis we generated homozygous G41S cytochrome *c* knock-in mice (*Cycs* ^G41S/G41S^) expressing mouse G41S cytochrome *c*. Surprisingly these mice have normal platelet counts and platelet formation. This has led us to discover an unexpected species specificity in the interaction between cytochrome *c* and Apaf-1, which has important implications for studies of the role of cytochrome *c* in the intrinsic apoptosis pathway.

## Materials and Methods

### CYC^G41S/G41S^ knock-in mice

All animal work complied with the Animal Welfare Act (1999) and was approved by the University of Otago Animal Ethics Committee at (AEC 57/10). Animals used in this study were euthanized by cervical dislocation. *Mus musculus* Linnaeus 1758 were modified by a single mutation (G41S) in the mouse somatic cytochrome *c* coding sequence. C57BL/6 mice heterozygous for the G41S mutation (*Cycs* ^+/G41S^) were generated by Taconic (Germany) and then bred to homozygosity. The presence of mouse G41S cytochrome *c* was confirmed by MALDI MS of cytochrome *c* purified from liver mitochondria of homozygous mice as previously described [13].

### Platelet depletion and counting

Platelet counts and platelet volume in mice were measured by obtaining blood via facial vein bleeding using a 5 mm animal lancet (Goldenrod). Blood was collected into EDTA coated 0.5 mL plastic tubes and counted using either the Cell-Dyn 3700 Hematology analyzer with veterinarian package (Abbott) or the Vetscan HM5 Hematology analyzer (Abaxis). Results are expressed ± SEM. Immune thrombocytopenia was induced in 5–6 month old mice by intravenous injection of anti-mouse thrombocyte (Cedarlane, CLA31440) as previously described [14]. The serum was diluted 1:25 in sterile PBS and 100 μL injected intravenously into the tail vein using aseptic technique.

### Analysis of hematopoietic compartments

After euthanasia single cell suspensions were prepared from thymus, spleen, inguinal lymph nodes or bone marrow. Cell number was determined using a hemocytometer. For determination of cellular composition cells were stained with fluorochrome-conjugated antibodies to CD4, CD8a, Ly6, CD11b, B220/CD45R, sIgM (BD Biosciences), and F4/80 (Serotec)), or isotype control antibodies, for 30 min and analysed by flow cytometry. Analysis was performed using FlowJo software (Tree Star Inc.).

### Expression and purification of recombinant proteins

Synthetic genes encoding mouse somatic wildtype (WT), mouse somatic G41S, mouse somatic A44P and mouse somatic G41S/A44P cytochrome *c* were purchased from Genscript and introduced into the pBTR expression vector. Cytochromes *c* were expressed and purified as previously described [15]. Cytochrome *c* concentrations were determined using ε_410_ = 0.1061 M^-1^cm^-1^ [16]. Recombinant human Apaf-1 was expressed as previously described [17]. Sf21 insect cells were cultured in Sf900III SFM Medium (Invitrogen). Recombinant baculovirus was used to infect 500 mL of cells at a density of 1 x 10^6^/mL. Cells were harvested 40 hours post infection, washed in cold phosphate buffered saline and re-suspended in 5 volumes of Buffer A (20 mM HEPES-KOH (pH 8.0), 10 mM KCl, 1.5 mM MgCl_2_, 1 mM EDTA, 1 mM ethylene glycol bis(2-aminoethyl ether) tetraacetic acid (EGTA), 0.1 mM dithiothreitol (DTT) and 1 mM phenylmethylsulfonyl fluoride (PMSF)). The cell suspension was incubated on ice for 1 hour and then lysed with a Dounce homogenizer. The cell lysate was clarified at 4°C by a two-step centrifugation procedure at 8,500 rpm for 60 min and 10,500 rpm for 10 min. The resultant supernatant was applied to a 5 mL HisTrap column (GE Healthcare) using an AKTA Prime FPLC protein purification system (GE Healthcare). The column was washed with Buffer A with 1 M NaCl and 20 mM imidazole followed by a stepwise elution with Buffer A supplemented with 250 mM imidazole. Eluted protein was dialysed into Buffer A and further purified on a MonoQ column (GE Healthcare) using an AKTA Purifier FPLC protein purification system (GE Healthcare). Apaf-1 was eluted using 0–1 M NaCl over 30 mL, dialysed back into Buffer A and stored at 4°C until required.

### Preparation of cytosolic extracts

Cytosolic extracts were prepared from a human monocytic cell line (U937) or from a mouse fibrosarcoma cell line (WEHI164) as previously described [18]. Briefly, cells were grown in RPMI 1640 medium with 10% fetal bovine serum in 5% CO_2_ at 37°C. Cells were collected and resuspended at a concentration of 8 × 10^7^ cells/mL in ice-cold cell extraction buffer (20 mM HEPES–KOH (pH 7.5), 10 mM KCl, 1.5 mM MgCl_2_, 1 mM EDTA, 1 mM EGTA, 1 mM DTT, 250 μM PMSF, and Complete protease inhibitor cocktail (Roche)). Cells were allowed to swell on ice for 60 min and were lysed by passing through a 28-gauge needle. Lysates were centrifuged for 10 min at 10,000 *g*, followed by ultracentrifugation at 186,000 *g* for 60 min. The protein concentration of the cytosolic extracts was determined using the BCA protein assay. Cytosolic extracts were stored in aliquots at -80°C until required.

Cytosolic extracts were prepared from Xenopus oocytes as previously described [19]. The jelly coat was removed from the oocytes by swirling in 2% cysteine (pH 7.8) for 5 min then washed 3 times in Marc’s Modified Ringers (MMR) solution (100 mM NaCl, 2 mM KCl, 1 mM MgSO_4_, 2.5 mM CaCl_2_, 5 mM HEPES-KOH (pH 7.6) and 80 μM EDTA) followed by 3 washes in Egg Lysis Buffer (ELB) (250 μM MgCl_2_, 5 mM KCl, 1 mM HEPES-KOH pH 7.7, 25 mM sucrose and 1 mM DTT). Oocytes were packed by centrifugation at 400 *g* for 15 sec. Excess ELB was removed and cylohexamine (178 μM), leupeptin (12 μM) and cytochalasin B (10.4 μM) added. Oocytes were then centrifuged for 12 min at 12,000 *g*, crude extract removed and subjected to centrifugation for 70 min at 186,007 *g*. The clear upper phase was recentrifuged for 25 min at 186,007 *g*. Xenopus cytosolic extracts were stored at -80°C until required.

### Caspase assays

Caspase assays were carried out as previously described [5,20]. For mammalian cells, purified recombinant cytochrome *c* was added to cytosolic extracts (100 μg protein) in the presence of 1 mM dATP in a final volume of 60 μL caspase assay buffer (100 mM HEPES–KOH (pH 7.25), 10% w/v sucrose, 0.1% w/v CHAPS, and 5 mM DTT) and incubated at 37°C. Xenopus cytosolic extracts (2 μg) were incubated at 37°C with purified recombinant cytochrome *c* and 1 mM ATP in a final volume of 30 μL caspase assay buffer for 30 min to preform apoptosomes. After a 30 min preincubation to form apoptosomes, samples were adjusted to a final volume of 60 μL using caspase assay buffer. Caspase 3-like activity was measured fluorimetrically by the addition of 50 μM acetyl-Asp- Glu-Val-Asp-7-amino-4-methylcoumarin (Ac-DEVD-AMC) at 37°C in 96-well plates using a POLARstar OPTIMA microplate reader (BMG LABTECH). The production of AMC (λ_EM_ = 390 nm, λ_EX_ = 460 nm) was assayed over 120 minutes and was quantified by reference to an AMC standard curve. The rate of caspase activation was calculated from the maximum slope of the progress curve.

### Statistics

Changes in variables were analysed by ANOVA with Tukey’s post-hoc test for multiple comparisons (Instat 3.1a) or Log-rank (Mantel-Cox) test (Prism 6). Differences were considered significant at *P* < 0.05.

## Results and Discussion

### Platelet counts and formation are normal in *Cycs* ^G41S/G41S^ mice

**Fig 1:**
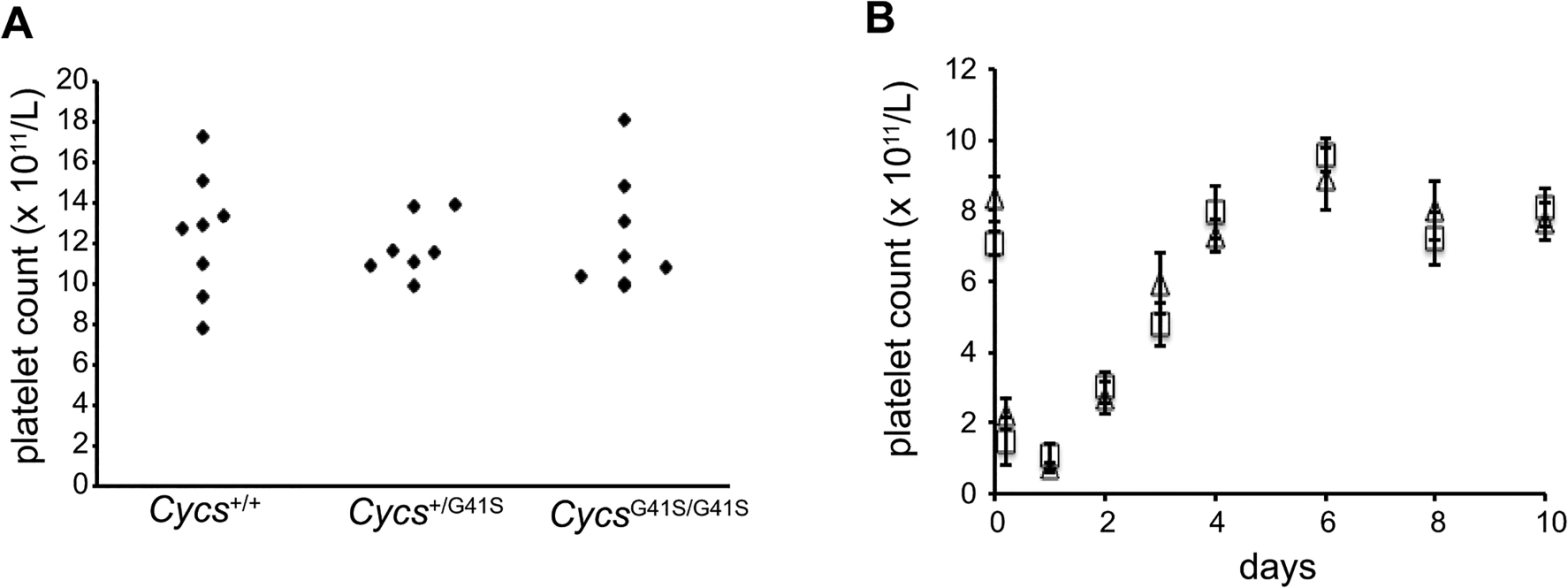
Platelet counts and formation are normal in *Cycs* ^G41S/G41S^ mice. A. Platelet counts of 8 week old *Cycs* ^**+/+**^, *Cycs* ^**+/G41S**^ and *Cycs* ^**G41S/G41S**^ mice. B. Anti-platelet serum was injected into *Cycs* ^**+/+**^ mice (□, n = 7–11) and *Cycs* ^**G41S/G41S**^ mice (△, n = 8–13) to deplete platelets and platelet count was monitored over 10 days. Data are presented as mean ± SEM.

The role of the intrinsic apoptosis pathway in platelet formation is controversial [21]. The thrombocytopenia phenotype associated with proapoptotic cytochrome *c* mutations [1,2] suggests that cytochrome *c* mediated caspase activation may be essential for platelet formation in humans. To investigate the molecular basis of Thrombocytopenia Cargeeg we generated a knock-in mouse expressing mouse G41S cytochrome *c* in place of mouse somatic cytochrome *c*. Homozygous *Cycs* ^G41S/G41S^ mice were born at the expected Mendelian frequency from heterozygous *Cycs* ^+/G41S^ breeding pairs, and had weight gain and lifespan indistinguishable from wildtype *Cycs* ^+/+^ mice (S1 Fig). Platelet counts did not differ between the three genotypes (Fig 1A), and there was no significant difference in mean platelet volume (6.8±0.1 fL (*Cycs* ^+/+^), 6.7±0.1 fL (*Cycs* ^+/G41S^), 6.9±0.2 fL (*Cycs* ^G41S/G41S^)). To determine whether there were any defects in platelet formation we monitored platelet recovery after induction of immune thrombocytopenia. As previously reported [14], intravenous injection with anti-platelet serum results in a severe thrombocytopenia within 5 h. Platelet count then returns to normal by 8–10 days. There was no difference in platelet production between *Cycs* ^+/+^ and *Cycs* ^G41S/G41S^ mice (Fig 1B).

### Mouse G41S cytochrome *c* has decreased caspase inducing activity *in vitro* but no effect on hematopoiesis

**Fig 2:**
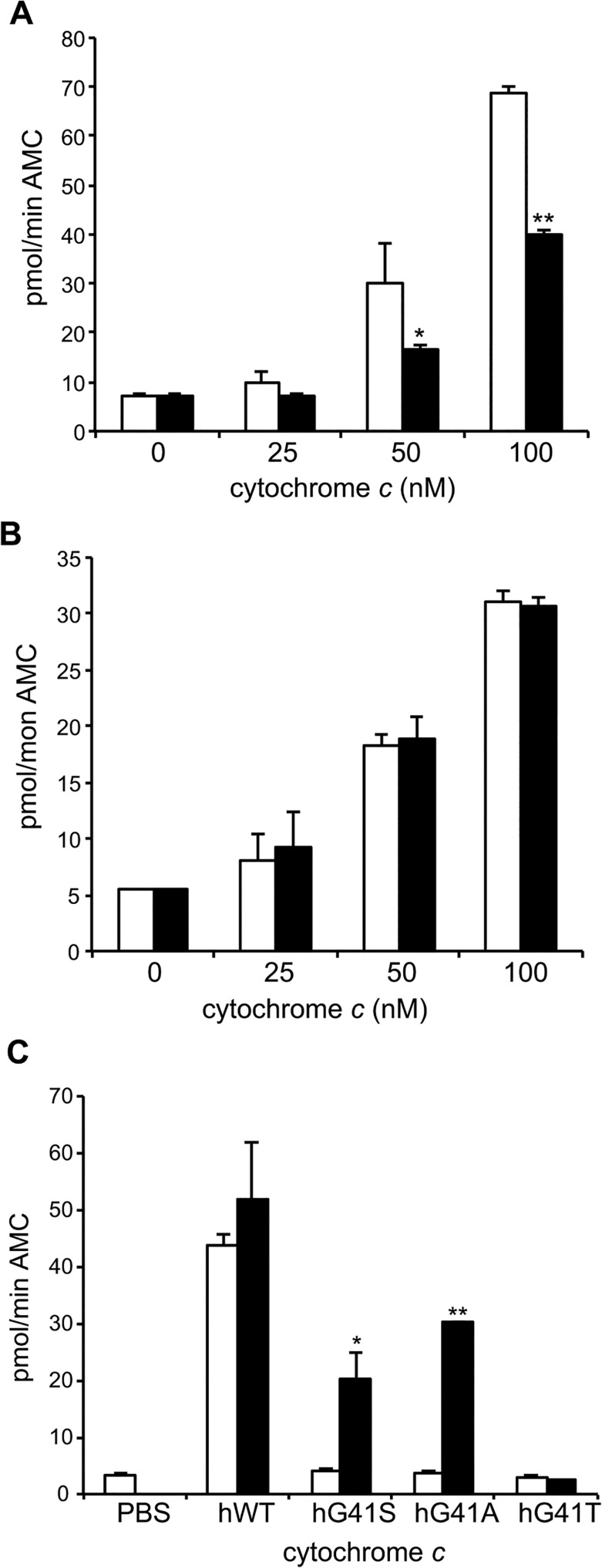
Mutations at residue 41 have species-specific effects on caspase activation. A. and B. Cleavage of the caspase 3 substrate Ac-DEVD-AMC was monitored in A. mouse WEHI164 (100 μg) and B. human U937 (100 μg) cytosols with the addition of 0 nM, 25 nM, 50 nM or 100 nM mouse somatic WT (white bars) or G41S (black bars) cytochrome *c* at pH 7.25. All reactions contained 1 mM dATP. Data are presented as mean ± SD (n = 3). *P < 0.01, **P < 0.001 compared to WT. C. Cleavage of the caspase 3 substrate Ac-DEVD-AMC was monitored in Xenopus cytosolic extracts (2 μg) with the addition of 100 nM human cytochrome *c* without (white bars) or with (black bars) the addition of human Apaf-1. Data are presented as mean ± SD (n = 3). *p < 0.01, **p < 0.001 compared to without human Apaf-1. Progress curves are shown in S2B–S2D Fig).

There were two possible explanations for the difference in phenotype between Thrombocytopenia Cargeeg subjects and the *Cycs* ^G41S/G41S^ knockin mice. There are differences in platelet formation between humans and mice, with mice producing a higher number of smaller platelets [22]. Thus the role of cytochrome *c* in platelet formation may not be conserved between humans and mice. In keeping with this there is substantial evidence that neither the intrinsic nor extrinsic apoptosis pathway is necessary for platelet formation in mice [12,21]. Alternatively the functional effect of the G41S mutation may not be conserved between human and mouse cytochrome *c*. To investigate the latter explanation, the activities of WT and G41S somatic mouse cytochromes *c* were assayed using cytosolic extracts obtained from either mouse or human cell lines (Fig 2). We have previously reported that human G41S cytochrome *c* has up to 2-fold increased caspase-inducing activity compared to human WT cytochrome *c* [1,5] (S2A Fig). In contrast here we observed that mouse G41S cytochrome *c* had 2-fold reduced caspase-inducing activity compared to mouse WT cytochrome *c* in mouse cytosolic extracts (Fig 2A) although interestingly the same activity as mouse WT cytochrome *c* in human cytosolic extracts (Fig 2B). In contrast de Rocco *et al*. reported that *Cycs* ^-/-^/*Cyct* ^-/-^ mouse lung fibroblasts expressing mouse G41S cytochrome *c* were significantly more sensitive to activation of the intrinsic apoptosis pathway compared to *Cycs* ^-/-^/*Cyct* ^-/-^ mouse lung fibroblasts expressing WT mouse cytochrome *c* [2]. The reason for the discrepancy between the results with recombinant protein and transduced cell lines is unclear. However the absence of thrombocytopenia in the CYC^G41S/G41S^ mice supports our finding of lack of functional conservation of the proapoptotic effect of the G41S mutation from human to mouse cytochrome *c*.

Knockout of the apoptotic function of cytochrome *c* results in embryonic or early post-natal lethality [23]. However diminished activation of the intrinsic apoptosis pathway causes abnormalities in the cellularity and composition of hematopoietic compartments [24,25]. To investigate whether the decreased apoptotic activity of mouse G41S cytochrome *c* affected hematopoiesis, the thymus, spleen, bone marrow and lymph nodes from *Cycs* ^+/+^ and *Cycs* ^G41S/G41S^ mice at 6–7 and 55–56 weeks were analysed. There were no differences in either total cellularity (S3 Fig) or cell composition (S1 Table) in any tissue examined. Together these results demonstrate that the presence of a mutant cytochrome *c* with a mildly reduced ability to activate caspases does not result in a discernible “loss of apoptosis” phenotype.

### Mutations in cytochrome *c* have species-specific effects on caspase activation

The previous result demonstrated that the functional impact of the G41S cytochrome *c* mutation was dependent on the species of both the cytochrome *c* and the Apaf-1. To extend this observation we made use of Xenopus oocyte cytosolic extracts, which have often been used to study the activation of the intrinsic apoptosis pathway [26]. We have previously shown that mutation of glycine 41 in human cytochrome *c* to alanine has no impact on caspase activation whereas mutation to threonine inhibits caspase activation, when tested in human cytosolic extracts [5]. As previously reported, human cytochrome *c* induces caspase activity in Xenopus extracts (Fig 2C), confirming that human cytochrome *c* binds to Xenopus Apaf-1. However none of the three residue-41 human cytochrome *c* variants induced caspase activity in Xenopus extracts (Fig 2C). This result implied that mutation of residue 41 blocked binding of human cytochrome *c* to Xenopus Apaf-1, therefore preventing subsequent caspase activation. To determine if this was the case, recombinant human Apaf-1 was added to the Xenopus extract. The addition of hApaf-1 rescued the ability of hG41S and hG41A, but not hG41T cytochrome *c*, to activate caspases (Fig 2C). These results are consistent with the activities of these variants in human cytosolic extracts [5] and thus confirm the role of species-specific interactions in the binding of cytochrome *c* to Apaf-1.

### Understanding the species-specific activation of caspases by G41S cytochrome *c*

**Fig 3:**
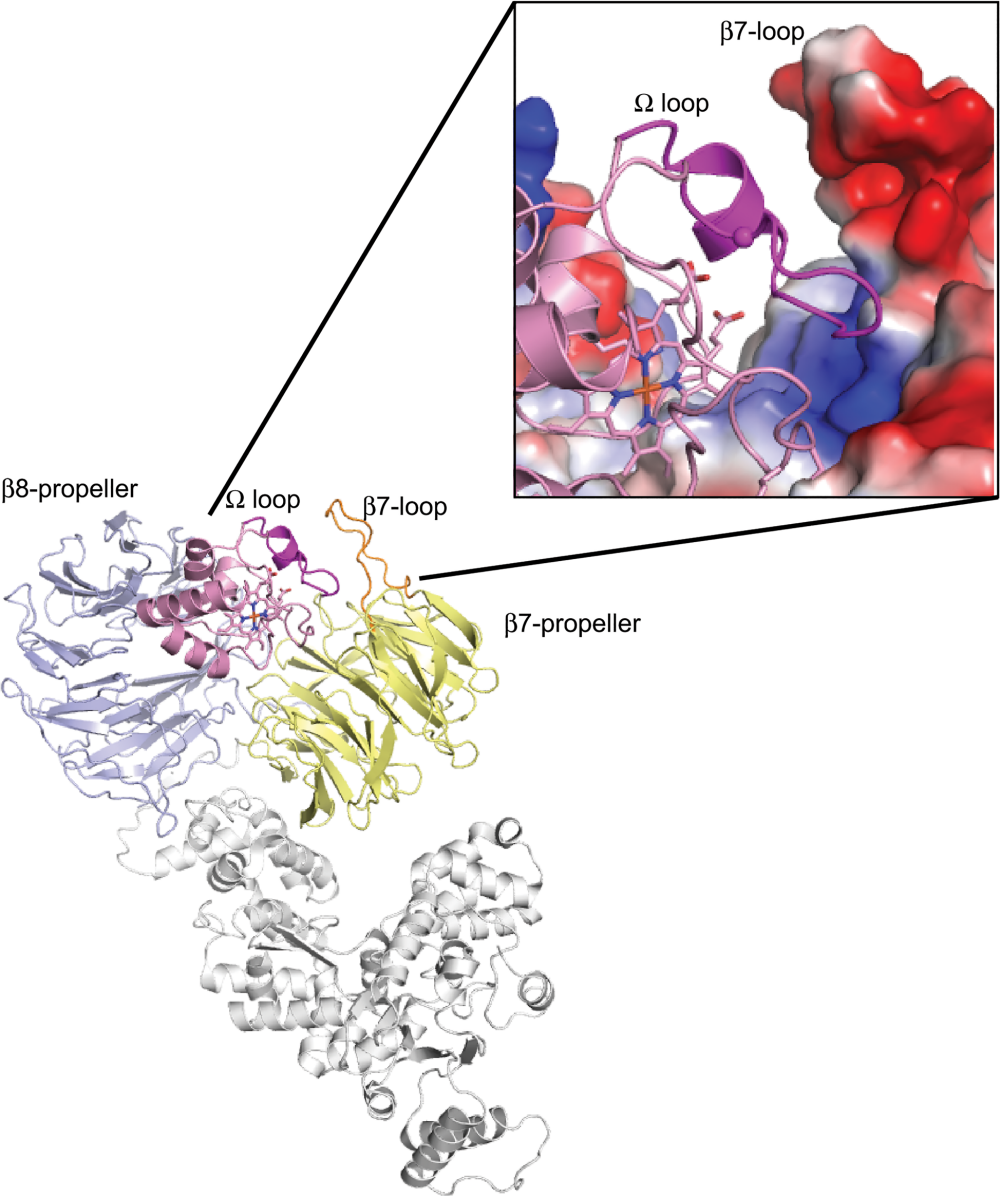
Interaction between cytochrome *c* and Apaf-1 in a human apoptosome model. Apaf-1 in grey (NBD, HD1, HD2 and WHD), pale blue (8 blade ß-propeller), lemon (7 blade ß-propeller) with the ß7 loop in orange. Cytochrome *c* in salmon-pink with the 40-to-57 Ω loop in magenta. Inset shows expansion of the cytochrome *c* 40-to-57 Ω loop with Gly-41 as a sphere and Apaf-1 as an electrostatic surface. Drawn from Protein Data Bank entry 3J2T.

**Fig 4:**
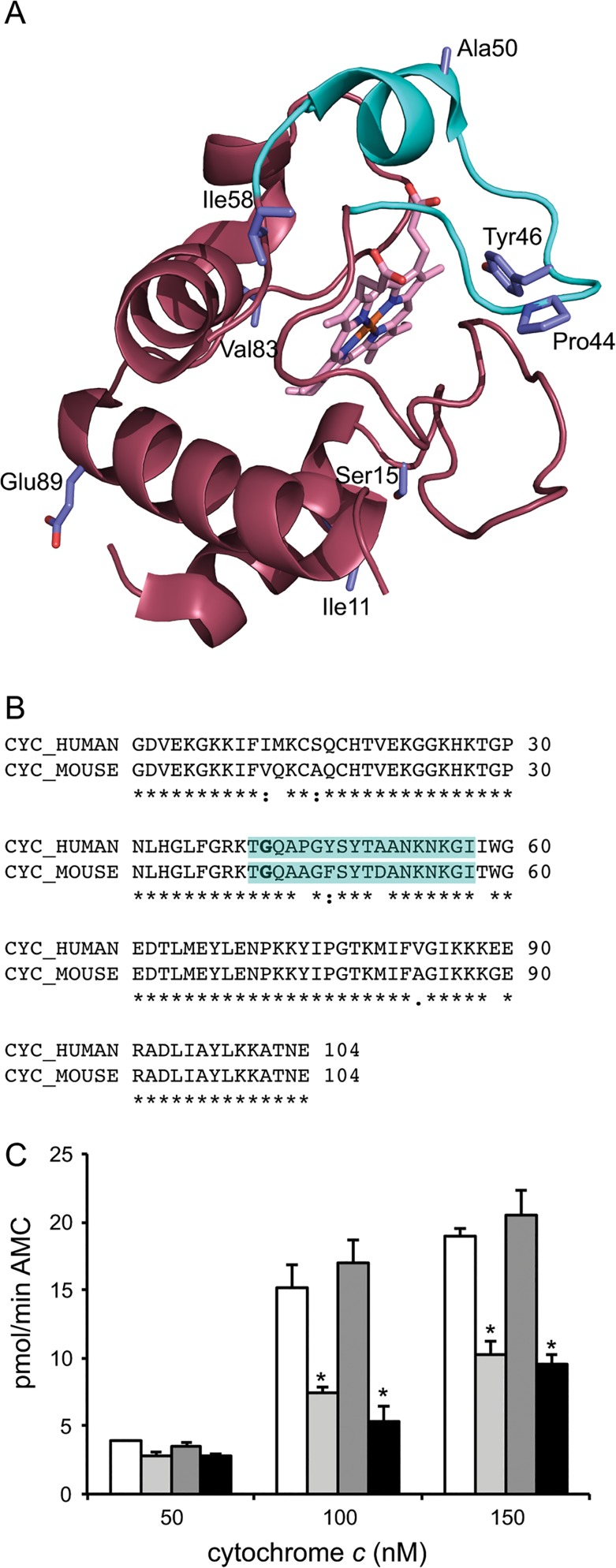
Variation at residue 44 does not alter the functional impact of the G41S mutation in mouse somatic cytochrome *c*. A and B. Human cytochrome *c* and mouse somatic cytochrome *c* differ in sequence at surface exposed residues. A. Human ferricytochrome *c* with side chains of residues differing between human and mouse somatic shown as sticks with carbon in pale blue, oxygen in red and nitrogen in blue. The backbone of the 40-to-57 Ω loop is in cyan. Drawn from Protein Data Bank entry 3NWV. B. Clustal Omega sequence alignment of human (P99999) and mouse somatic (P62897) cytochromes *c* with the initiating Met removed. The residues comprising the 40-to-57 Ω loop are in cyan and Gly41 is in bold. C. Cleavage of the caspase 3 substrate Ac-DEVD-AMC was monitored in mouse WEHI164 (100 μg) cytosols with the addition of 50 nM, 100 nM or 150 nM of mouse somatic WT (white bars), G41S (light grey bars), A44P (dark grey bars) or G41S/A44P (black bars) cytochrome *c*. The rate in the absence of cytochrome *c* was 2.74 ± 0.24 pmol/min AMC. Data are presented as mean ± SD (n = 3). *P < 0.001 compared to WT. Progress curves are shown in S4 Fig.

Cytochrome *c* interacts with the C-terminal WD40 domain of Apaf-1, binding in a grove between the 7- and 8-blade ß-propellers (Fig 3). This interaction is predominantly driven by electrostatic interactions between surface lysine residues of cytochrome *c* and aspartic acid residues of Apaf-1 [27]. The difference in functional impact of the G41S mutation between mouse and human was therefore unexpected since the two cytochromes *c* share 91.4% sequence identity (Fig 4A), including conservation of the surface lysine residues. Furthermore given the minimal impact of the G41S mutation on the structure of cytochrome *c* [15], the electrostatic interactions are unlikely to be altered by the change from glycine to serine.

Our results suggest that the 40-to-57 Ω-loop, which encompasses both naturally occurring cytochrome *c* mutations (G41S [1] and Y48H [2]), contributes to determining both the affinity of the cytochrome *c* interaction and the species specificity of the interaction. Three of the nine residue differences between human and mouse cytochromes *c* are located on this loop: P44A, Y46F and A50D (Fig 4A), and these residues also vary in Xenopus cytochrome *c* (E44, F46 and D50). We hypothesized that the variation in this loop could contribute to the contrasting effects of the G41S mutation in mouse and human cytochromes *c*. We focused on residue 44 since an alignment of the structures of human and bovine ferricytochromes *c* (S5 Fig) shows that the 40-to-57 Ω-loop main chain at this position is shifted between human (with P44, Y46 and A50) and bovine (which resembles human at residue 44 (P44) but mouse at residues 46 (F46) and 50 (D50)). In addition residues flanking residue 44 have been reported to undergo a conformational transition during apoptosis [28]. Mouse A44P cytochrome *c* has the same caspase-inducing activity as WT, and the activity of the mG41S/A44P variant was decreased to a similar extent as mG41S cytochrome *c* (Fig 4C). Thus the identity of residue 44 does not explain the difference in effect of the G41S mutation between human and mouse cytochromes *c*. Instead variation in the Apaf-1 sequence at the interacting surface may explain the difference in the effect of the G41S mutation in mouse and human Apaf-1.

**Fig 5:**
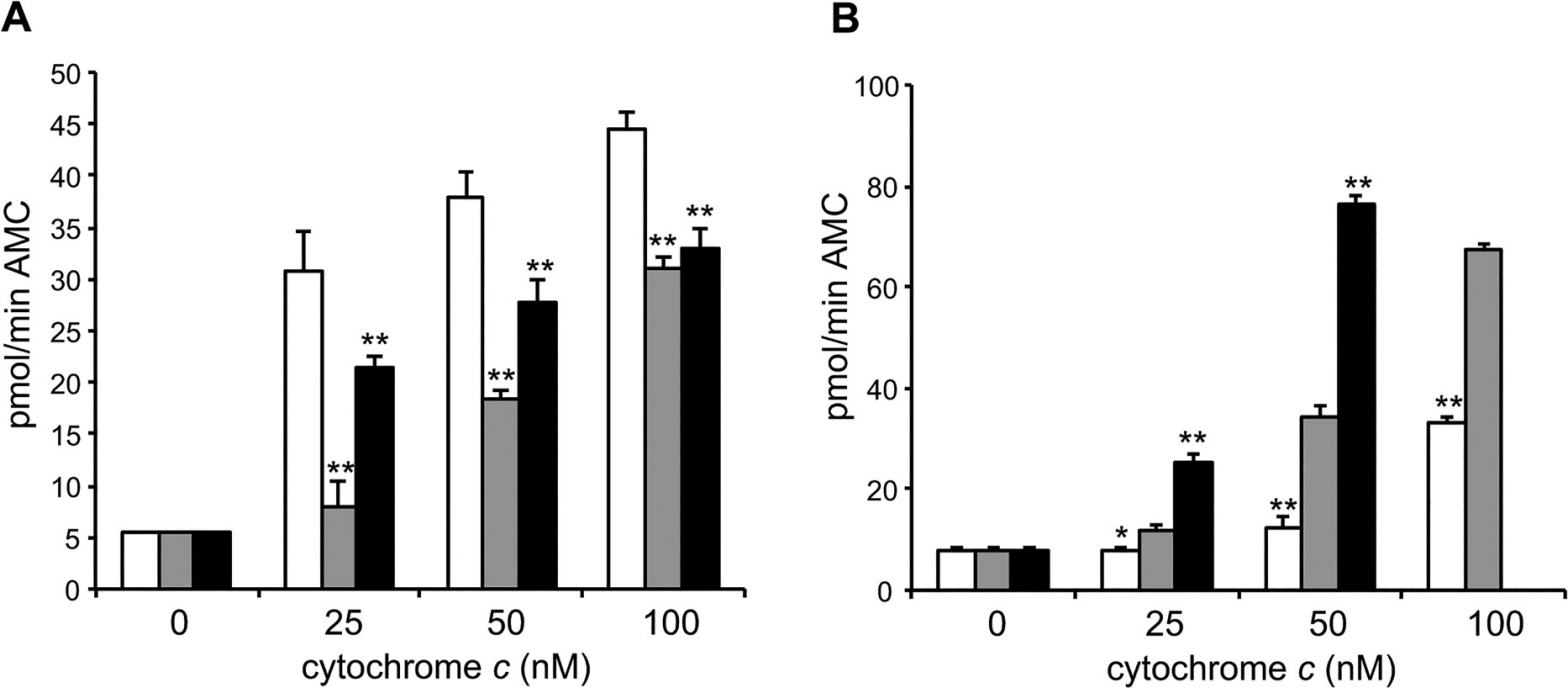
Cytochromes c from different specifies have different abilities to activate caspases. Cleavage of the caspase 3 substrate Ac-DEVD-AMC was monitored in human U937 (100 μg, A) and mouse WEHI164 (100 μg, B) cytosols with the addition of 0 nM, 25 nM, 50 nM or 100 nM human (white bars), mouse somatic (grey bars) or horse (black bars) cytochrome *c*. Data are presented as mean ± SD (n = 3). *P < 0.05, **P < 0.001 compared to human (A) or mouse (B).

Taken together these results suggest the interaction between Apaf-1 and cytochrome *c* is modulated by factors additional to the electrostatic interaction. Kluck *et al*. reported that the ability of cytochromes *c* from a variety of metazoan species to activate caspases was maintained in cytosolic extracts from different species [18]. As a result the majority of studies of the cytochrome *c*–Apaf-1 interaction have used proteins from different species. However Chertkova *et al*., using mouse cytosol, found that mouse cytochrome *c* had greater caspase-inducing activity compared to human and horse cytochromes *c* [29]. We confirm and extend the conclusions of Chertkova *et al*, finding that the ability of wildtype cytochromes *c* to induce caspase activation varied between cytosolic extracts prepared from human and mouse cell lines (Fig 5). It is likely that the use of saturating cytochrome *c* concentrations by Kluck *et al*. [18] masked the species differences.

## Conclusion

Our results demonstrate that the well described electrostatic interaction between cytochrome *c* and Apaf-1 is modulated by additional species-specific factors. Based on our results we propose that this modulation is driven by the interaction of the 40-to-57 Ω loop in cytochrome *c* with Apaf-1. Modeling of the crystal structures of mouse Apaf-1 and bovine cytochrome *c* onto a cryo-microscopy structure of the apoptosome (comprising human Apaf-1, bovine cytochrome *c* and human procaspase-9) has provided a clear definition of the Apaf-1 surface where cytochrome *c* binds; however the orientation of cytochrome *c* within the binding groove is less certain [30]. In the modeled apoptosome structure, the 40-to-57 Ω loop of cytochrome *c* is in close proximity to the ß7 loop that connects the d-strand of blade 4 with the a-strand of blade 5 in the 7-blade Apaf-1 ß-propeller [30] (Fig 3). However precise identification of Apaf-1 residues that are potentially important for an interaction with the 40-to-57 Ω loop of cytochrome *c* is not possible as the ß7 loop is not resolved in the mouse Apaf-1 crystal structure used to develop the apoptosome model [31]. This lack of resolution suggests flexibility of the ß7 loop, which may move to accommodate cytochrome *c* binding. Interestingly the ß7 loop is two residues shorter in Xenopus compared to that in eight different vertebrates (S6 Fig) supporting variation in this loop as a contributing factor for the observed species-specific effects of cytochrome *c* mutations on Apaf-1 interaction.

Based on the many reported examples in which cytochrome *c* from one species has been used in cytosolic extracts from another species it appears there is a general assumption that the species of cytochrome *c* used is unimportant. Our finding of both a species-dependent interaction of cytochrome *c* with Apaf-1, and a species-specific effect of cytochrome *c* mutations, is an important consideration for future *in vitro* and *in vivo* experiments that investigate cytochrome *c*-mediated apoptosis.

## Supporting Information

S1 Fig Weight and survival of *Cycs* ^G41S/G41S^ mice. A. Weights of *Cycs* ^+/+^ (x, n = 14), *Cycs* ^+/G41S^ (○, n = 15) and *Cycs* ^G41S/G41S^ (△, n = 15) mice. Data are presented as mean ± SEM. B. Kaplan Meier survival curve for *Cycs* ^+/+^, *Cycs* ^+/G41S^ and *Cycs* ^G41S/G41S^ mice.
(EPS)

S2 Fig The G41S mutation has opposite effects on the ability of human and mouse cytochrome c to activate caspases. A. Cleavage of the caspase 3 substrate Ac-DEVD-AMC was monitored in human U937 cytosols (100 μg) with the addition of 25 nM human WT (hWT), human G41S (hG41S), mouse somatic WT (mWT) or mouse somatic G41S (mG41S) cytochrome *c* at pH 7.25. All reactions contained 1 mM dATP. Data are presented as mean ± SD (n = 3). *P < 0.05 compared to WT; ns, not significant. B-D. Progress curves for caspase activity data in Fig 2A–2C.
(EPS)

S3 Fig Characterization of hematopoietic compartments in *Cycs* ^+/+^ and Cycs^G41S/G41S^ mice. Total cell count in lymph nodes, thymus, bone marrow and spleen isolated from young (5–6 weeks) and old (55–56 weeks) *Cycs* ^+/+^ (white bars, n = 7 young, n = 5 old and *Cycs* ^G41S/G41S^ (black bars, n = 9 young, n = 6 old) mice. Data are presented as mean ± SEM.
(EPS)

S4 Fig Variation at residue 44 does not alter the functional impact of the G41S mutation in mouse somatic cytochrome *c*. Progress curves for caspase activity data in Fig 4C. A. 50 nM cytochrome *c*, B. 100 nM cytochrome *c*, C. 150 nM cytochrome *c*.
(EPS)

S5 Fig Comparison of the variable 40-to-57 Ω loop residues in human and bovine cytochromes *c*. MacPyMOL Alignment of human (pink, PDB:3zcf) and bovine (cyan, PDB:2b42) ferricytochromes *c*. The loop is indicated in a darker shade with the side chains of the variable residues labeled and shown as sticks.
(EPS)

S6 Fig Clustal Omega alignment of Apaf-1 ß7 loop residues. Human (*Homo sapiens*, O14727), mouse (*Mus musculus*, O88879), rat (*Rattus norvegicus*, Q9EPV5), cow (*Bos taurus*, F1MUW4), horse (*Equus caballus*, F6R504), dog (*Canis familiaris*, J9NS21), Rhesus macaque (*Macaca mulatta*, XP_001086945.1), chicken (*Gallus gallus*, F1P1P3), zebrafish (*Danio rerio*, Q9I9H8*)*, frog (*Xenopus laevis*, Q6GNU6).
(PDF)

S1 Table Proportion of cell types in hematopoietic compartments of *Cycs* ^+/+^ and *Cycs* ^G41S/G41S^ mice. Cells were isolated from 5–6 week old mice and analysed by flow cytometry.
(DOCX)
