# Supplementary material for: Interspecies Variation in the Functional Consequences of Mutation of Cytochrome c
Source: PLoS One. 2015 Jun 18;10(6):e0130292. doi: 10.1371/journal.pone.0130292 (PMC4472513; doi:10.1371/journal.pone.0130292)
Supplement: S6 Fig — Human (Homo sapiens, O14727), mouse (Mus musculus, O88879), rat (Rattus norvegicus, Q9EPV5), cow (Bos taurus, F1MUW4), horse (Equus caballus, F6R504), dog (Canis familiaris, J9NS21), Rhesus macaque (Macaca mulatta, XP_001086945.1), chicken (Gallus gallus, F1P1P3), zebrafish (Danio rerio, Q9I9H8), frog (Xenopus laevis, Q6GNU6). (PDF) [file pone.0130292.s006.pdf]

CLUSTAL O(1.2.1) multiple sequence alignment

```
HUMAN             783  VKQFFLNLEDPQEDMEVIV  801
MOUSE                  VKRFFLSSEDPPEDVEVIV
RAT                    VKRFFLSSEDPPEDVEVIV
COW                    VKQFFLNSEEPQEDLEVIV
HORSE                  VKQFFISSEEPQEDMEVIV
DOG                    VKQFFLNSEEPQEDMEVIV
RHESUS MACAQUE         VKQFFLNSEDPQEDMEVIV
CHICKEN                IKDFFRNADEQQDDVEVLV
FROG                   VAKLFENEDESQ--PEVLL
ZEBRAFISH              VDSFFPESDE---EIKAMV
                       :  :* . ::     :.::
```
