# Supplementary material for: Interspecies Variation in the Functional Consequences of Mutation of Cytochrome c
Source: PLoS One. 2015 Jun 18;10(6):e0130292. doi: 10.1371/journal.pone.0130292 (PMC4472513; doi:10.1371/journal.pone.0130292)
Supplement: S1 Table — Cells were isolated from 5–6 week old mice and analysed by flow cytometry. (DOCX) [file pone.0130292.s007.docx]

|  | ***Cycs*^+/+^**  (%±SEM, n=5) | ***Cycs*^G41S/G41S^**  (%±SEM, n=7) |
| --- | --- | --- |
| **Bone Marrow** |  |  |
| Immature/mature B  (sIgM^+^/B220^+^) | 8.8 ± 1.1 | 5.9 ± 2.0 |
| ProB/PreB  (sIgM^-^/B220^+^) | 16.2 ± 3.2 | 16.2 ± 5.3 |
| Macrophages  (CD11b^+^/F480^+^) | 50.8 ± 2.5 | 42.7 ± 5.7 |
| Granulocytes  (CD11b^+^/Ly6^+^) | 45.7 ± 3.5 | 39.9 ± 1.6 |
| **Thymus** |  |  |
| Pro-T  (CD4^-^/CD8^-^) | 6.6 ± 3.9 | 14.1 ± 5.7 |
| Pre-T  (CD4^+^/CD8^+^) | 69.1 ± 6.1 | 68.4 ± 5.6 |
| Mature T  (CD4^+^/CD8^-^ or CD4^-^/CD8^+^) | 24.3 ± 4.4 | 17.5 ± 1.7 |
| **Spleen** |  |  |
| Naïve/mature B  (sIgM^+^/B220^+^) | 27.8 ± 3.8 | 23.3 ± 6.8 |
| Other B  (sIgM^-^/B220^+^) | 10.6±1.8 | 5.9±2.1 |
| Mature T  (CD4^+^/CD8^-^ or CD4^-^/CD8^+^) | 35.8±2.1 | 36.8±5.4 |
| Macrophages  (CD11b^+^/F480^+^) | 10.2 ± 0.9 | 8.2 ± 0.6 |
| Granulocytes  (CD11b^+^/Ly6^+^) | 10.7 ± 3.9 | 6.8 ± 0.6 |
| **Lymph node** |  |  |
| Naïve/mature B  (sIgM^+^/B220^+^) | 18.3 ± 2.7 | 14.8 ± 4.2 |
| Mature T  (CD4^+^/CD8^-^ or CD4^-^/CD8^+^) | 59.7 ± 2.8 | 48.5 ± 8.3 |
